# Supplementary material for: 3D Printing of a Self-Healing Thermoplastic Polyurethane through FDM: From Polymer Slab to Mechanical Assessment
Source: Polymers (Basel). 2021 Jan 19;13(2):305. doi: 10.3390/polym13020305 (PMC7835810; doi:10.3390/polym13020305)
Supplement: Supplementary file 1 [file polymers-13-00305-s001.pdf]

Supplementary Materials

3D printing of a self-healing thermoplastic polyurethane: from polymer slab to mechanical assessment

Linda Ritzen, Vincenzo Montano and Santiago J. Garcia*

Novel Aerospace Materials group, Faculty of Aerospace Engineering, Delft University of Technology, Kluyverweg 1, 2629 HS, Delft, The Netherlands; L.Ritzen@tudelft.nl (L.R.), V.Montano@tudelft.nl (V.M.)

* Correspondence: S.J.GarciaEspallargas@tudelft.nl (S.G.)

Compression cut test results

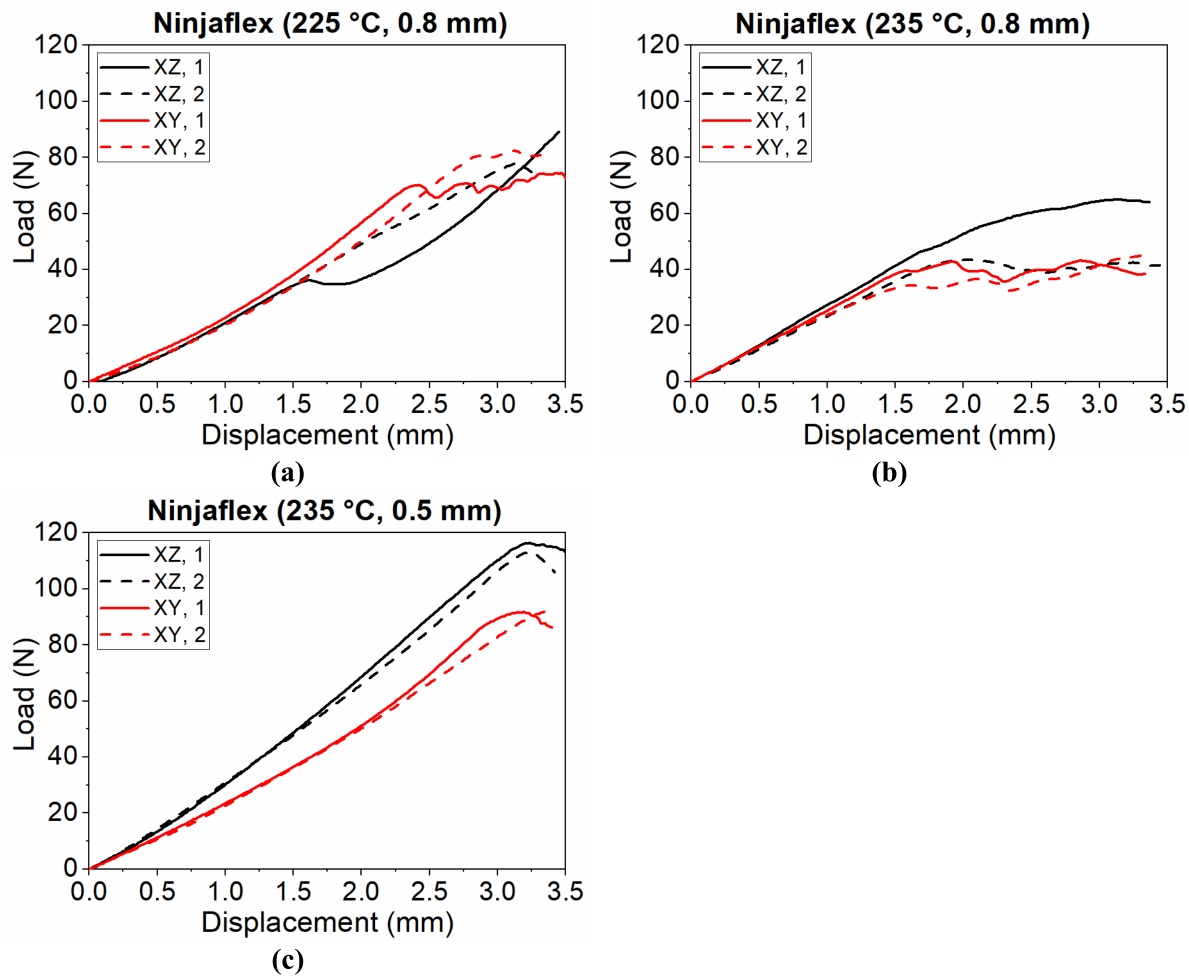

Figure S1. Force displacement curves of the compression cut test of all Ninjaflex samples. (a) 225 °C printing temperature and 0.8 mm infill distance. (b) 235 °C printing temperature and 0.8 mm infill distance. (c) 235 °C printing temperature and 0.5 mm infill distance.

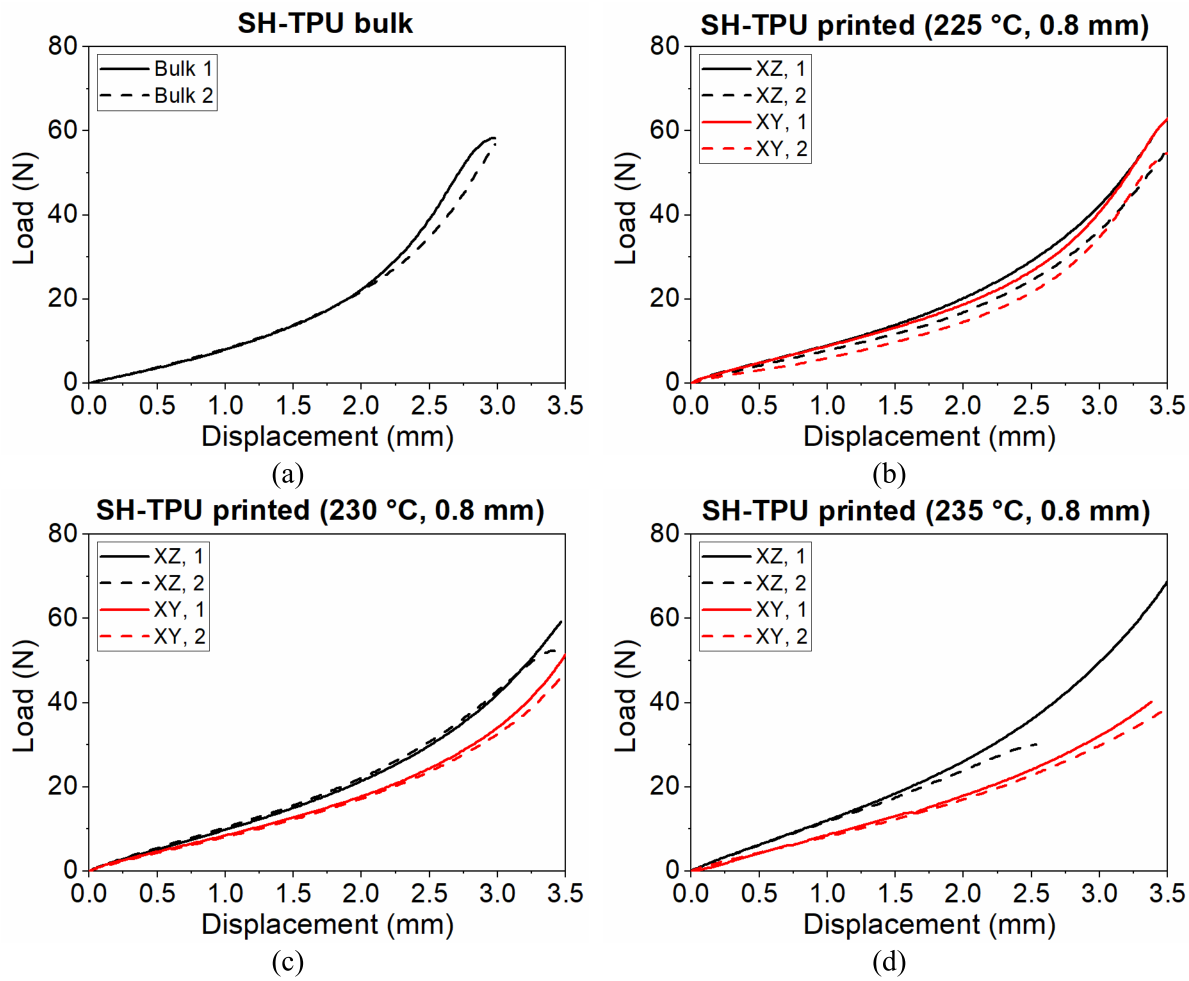

Figure S2. Force displacement curves of the compression cut test of all SH-TPU samples. (a) Bulk polymer. (b) 225 °C printing temperature and 0.8 mm infill distance. (c) 230 °C printing temperature and 0.8 mm infill distance. (d) 235 °C printing temperature and 0.8 mm infill distance.

NF_225_0.8_XZ_1

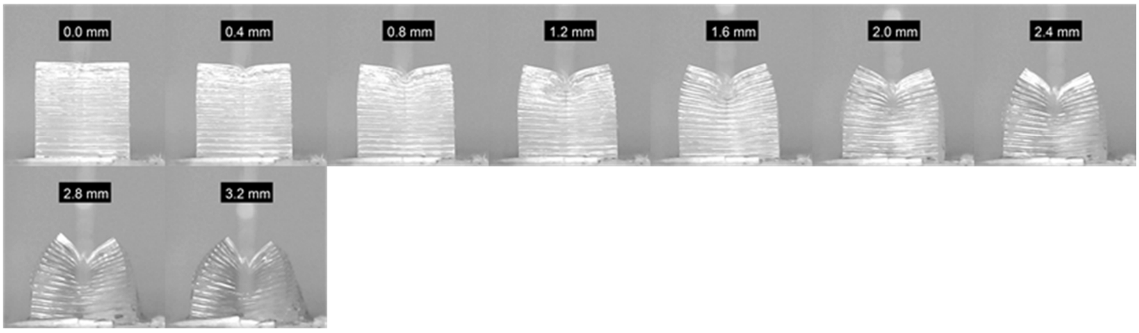

(a)

NF_225_0.8_XZ_2

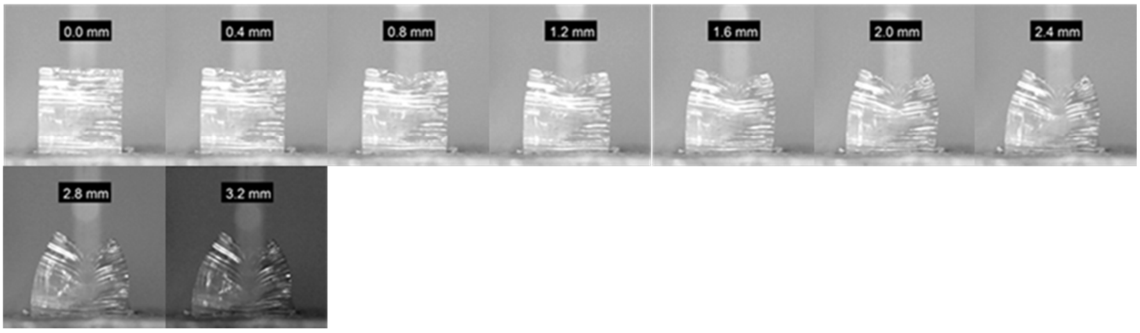

(b)

NF_225_0.8_XY_1

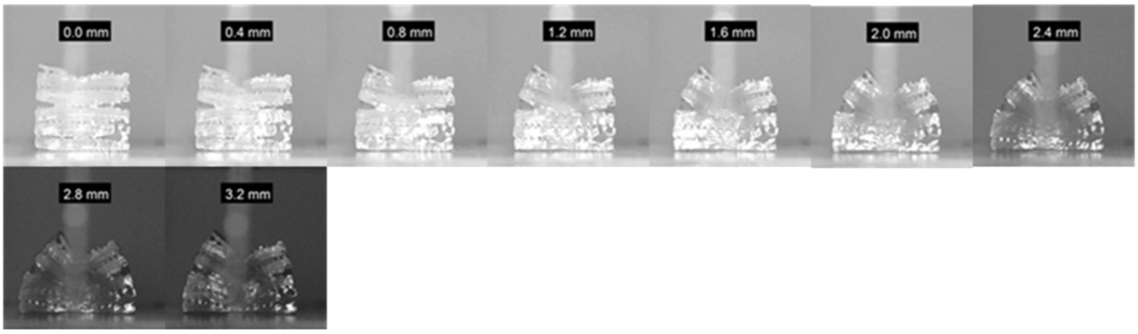

(c)

NF_225_0.8_XY_2

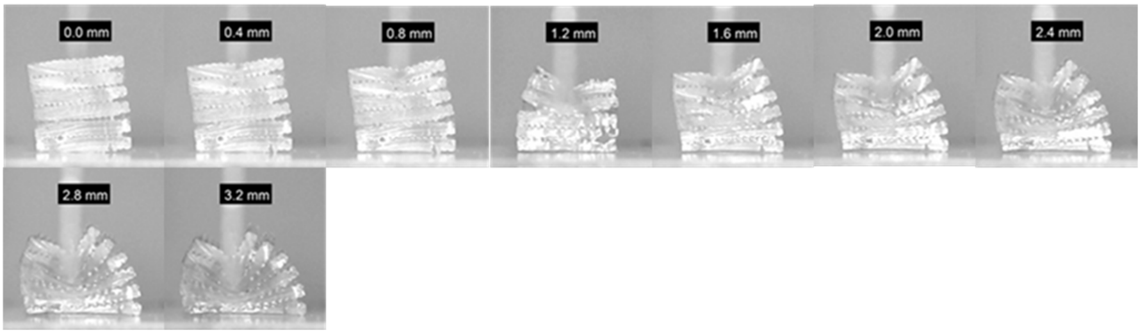

(d)

Figure S3. Micrographs taken during the compression cut test of the Ninjaflex sample printed at 225 °C printing temperature and 0.8 mm infill distance. (a) XZ sample 1. (b) XZ sample 2. (c) YZ sample 1. (d) YZ sample 2.

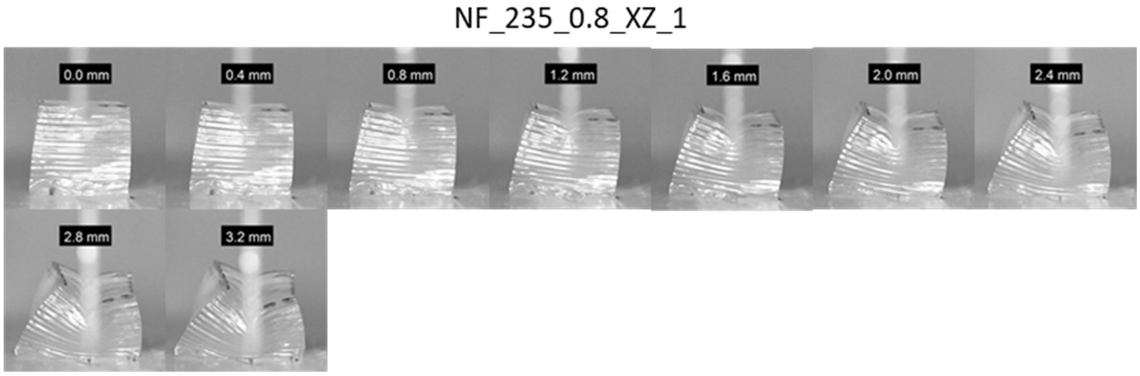

(a)

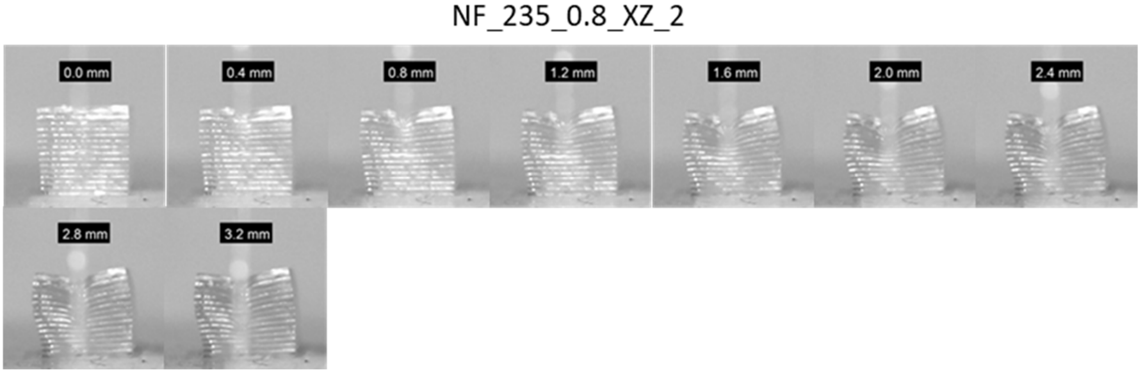

(b)

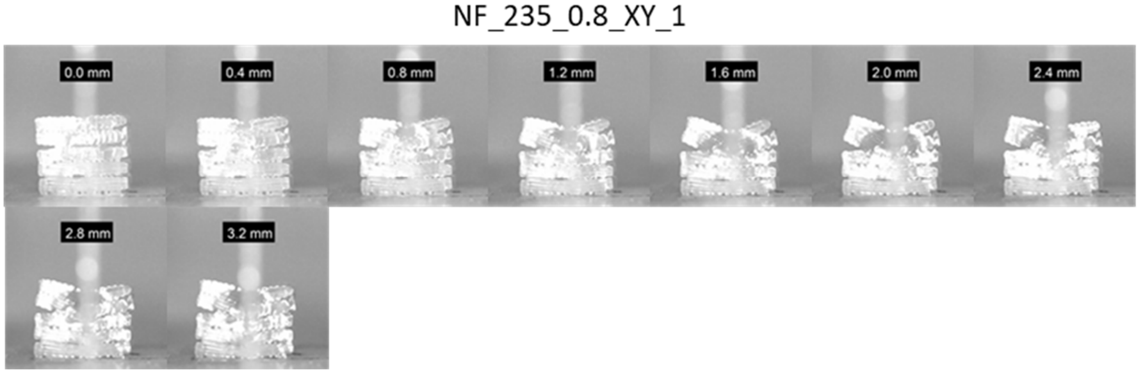

(c)

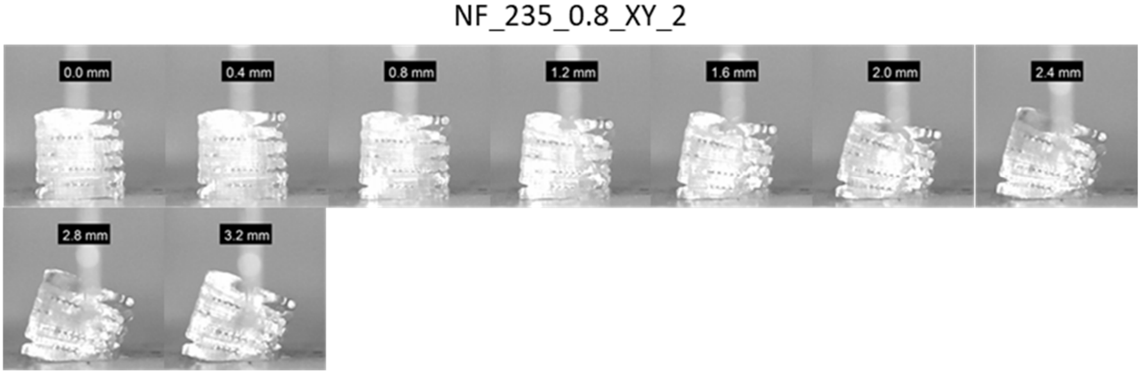

(d)

Figure S4. Micrographs taken during the compression cut test of the Ninjaflex sample printed at 235 °C printing temperature and 0.8 mm infill distance. (a) XZ sample 1. (b) XZ sample 2. (c) YZ sample 1. (d) YZ sample 2.

NF_235_0.5_XZ_1

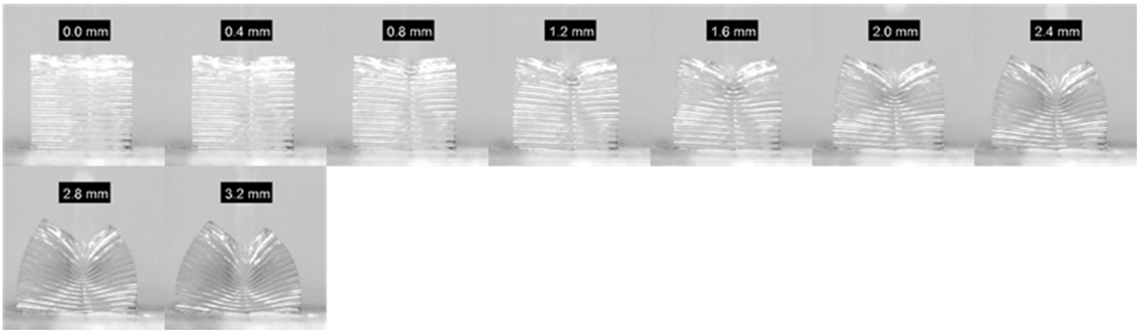

(a)

NF_235_0.5_XZ_2

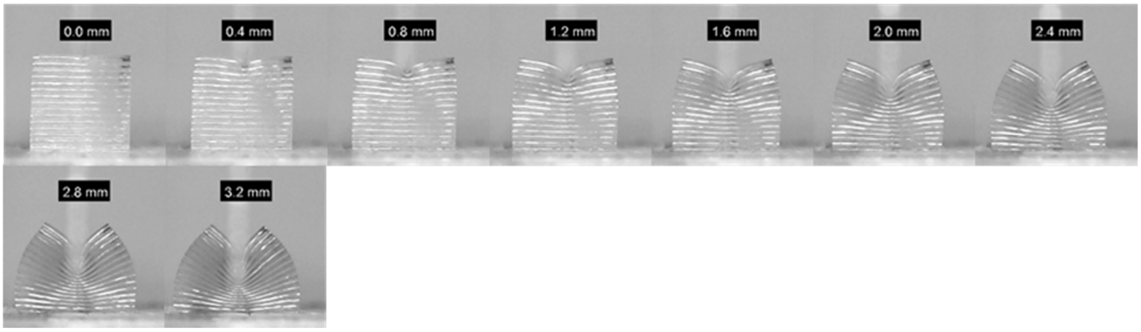

(b)

NF_235_0.5_XY_1

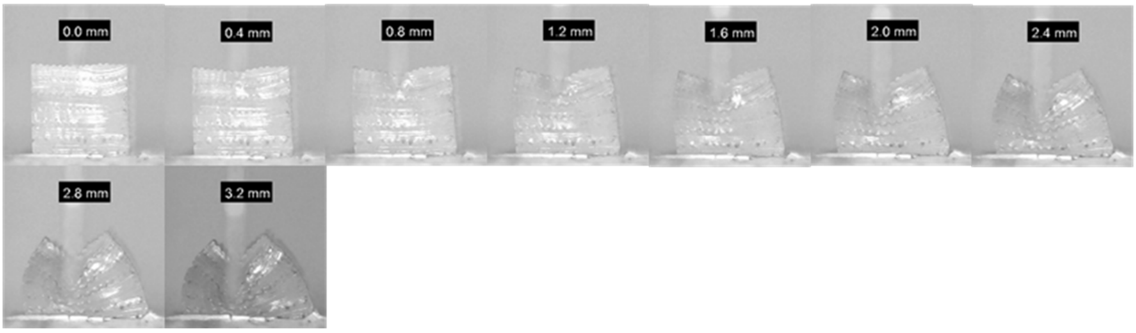

(c)

NF_235_0.5_XY_2

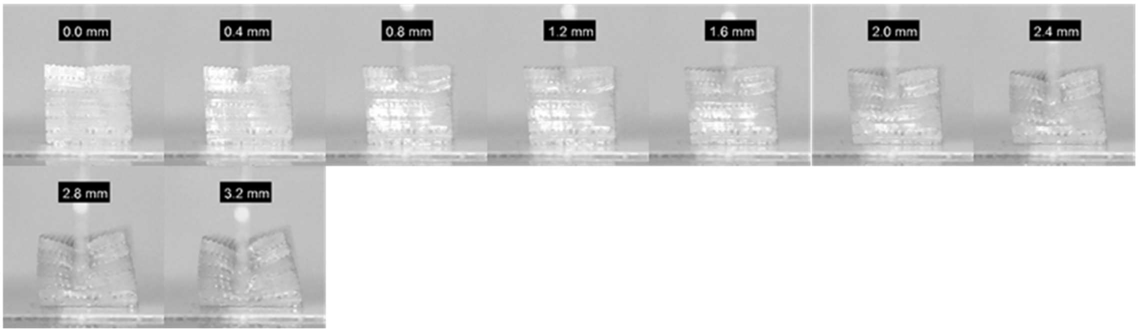

(d)

Figure S5. Micrographs taken during the compression cut test of the Ninjaflex sample printed at 235 °C printing temperature and 0.5 mm infill distance. (a) XZ sample 1. (b) XZ sample 2. (c) YZ sample 1. (d) YZ sample 2.

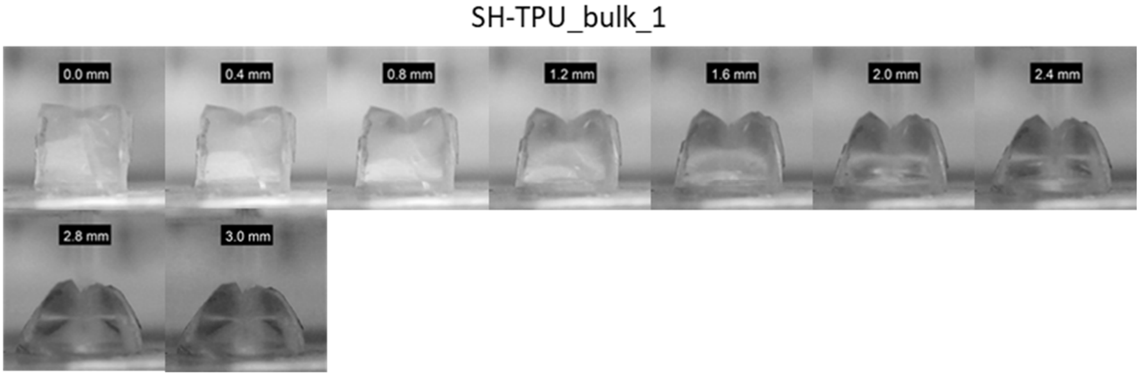

(a)

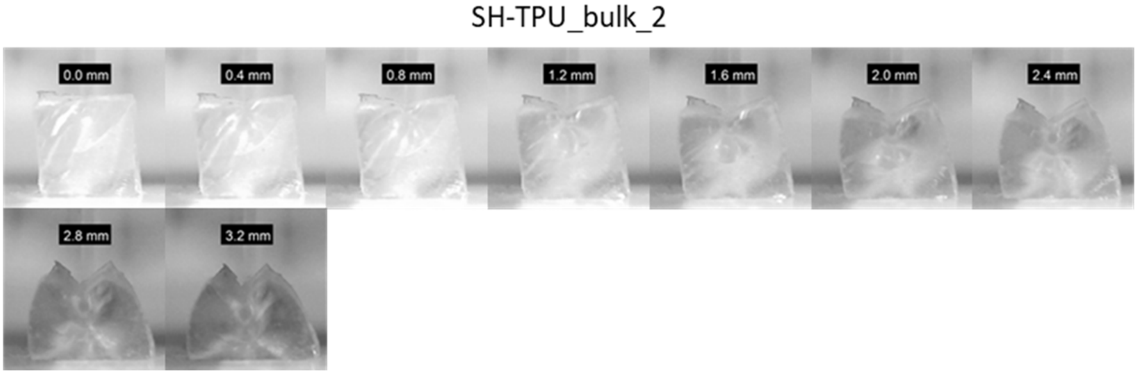

(b)

Figure S6. Micrographs taken during the compression cut test of bulk SH-TPU. (a) Sample 1. (b) Sample 2.

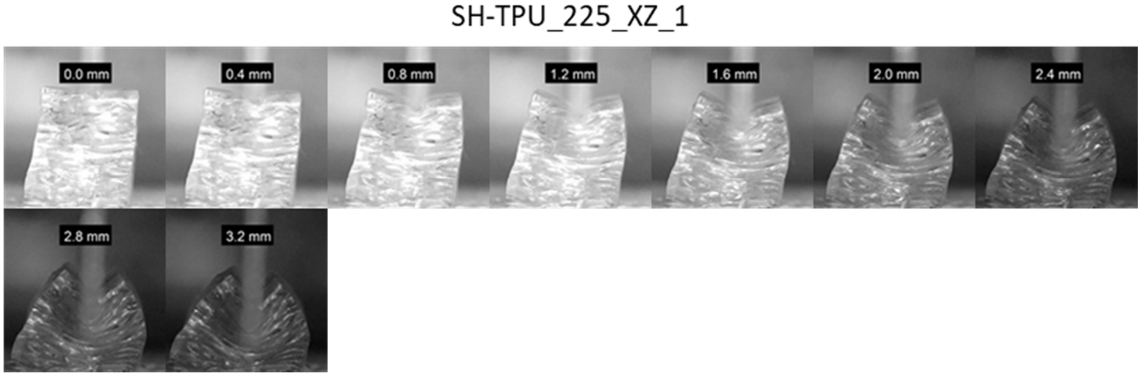

(a)

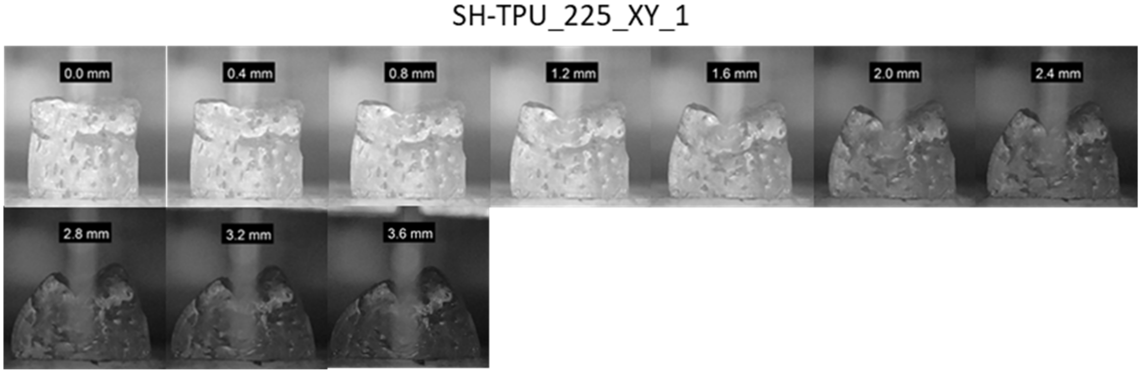

(b)

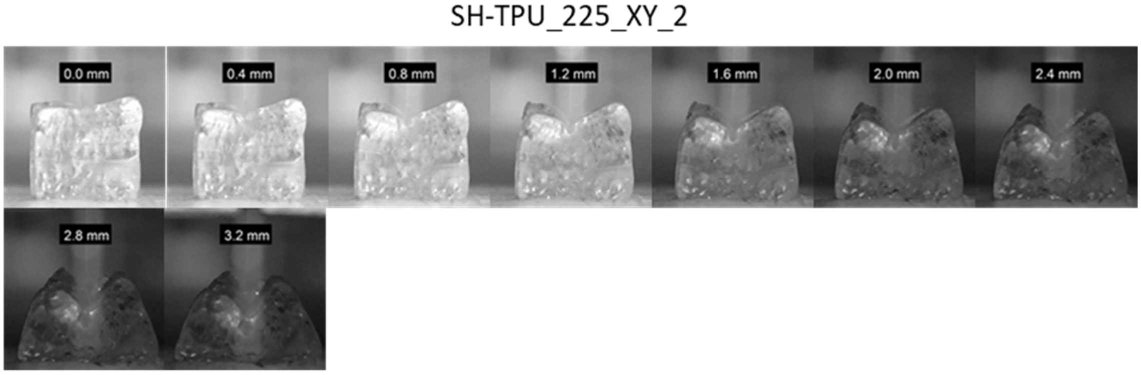

(c)

Figure S7. Micrographs taken during the compression cut test of the SH-TPU sample printed at 225 °C printing temperature and 0.8 mm infill distance. (a) XZ sample 1. (b) XY sample 1. (c) XY sample 2. (XZ sample 2 is missing)

SH-TPU_230_XZ_1

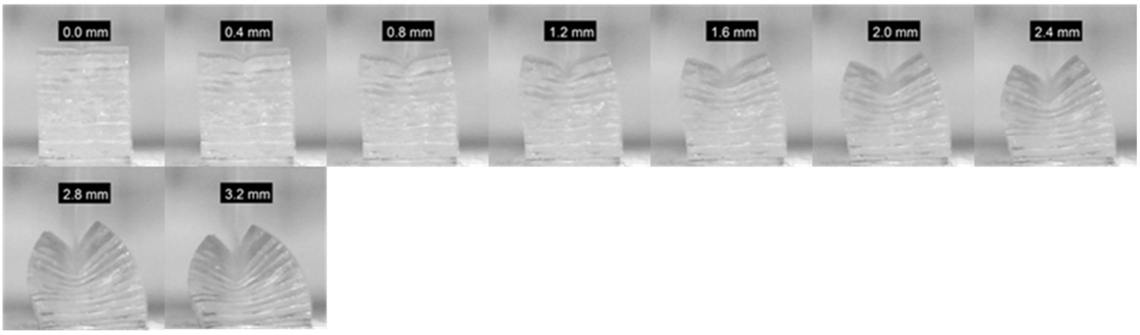

(a)

SH-TPU_230_XZ_2

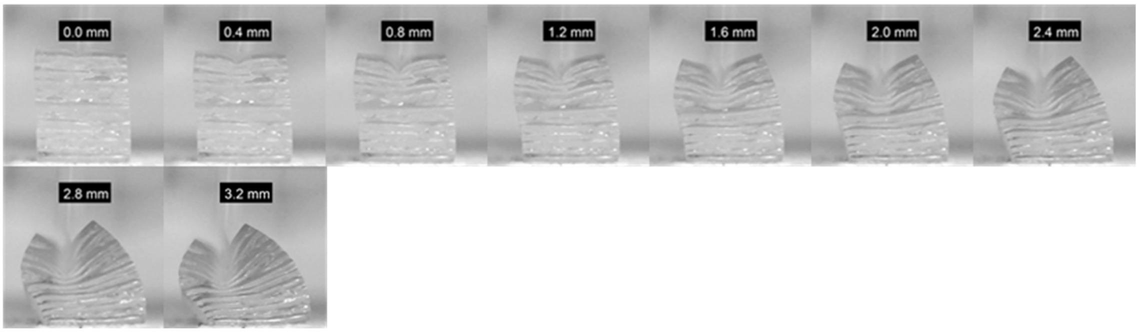

(b)

SH-TPU_230_XY_1

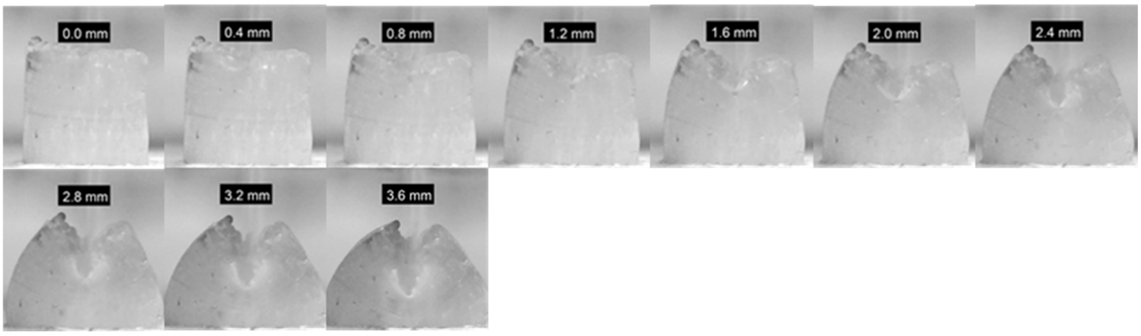

(c)

SH-TPU_230_XY_2

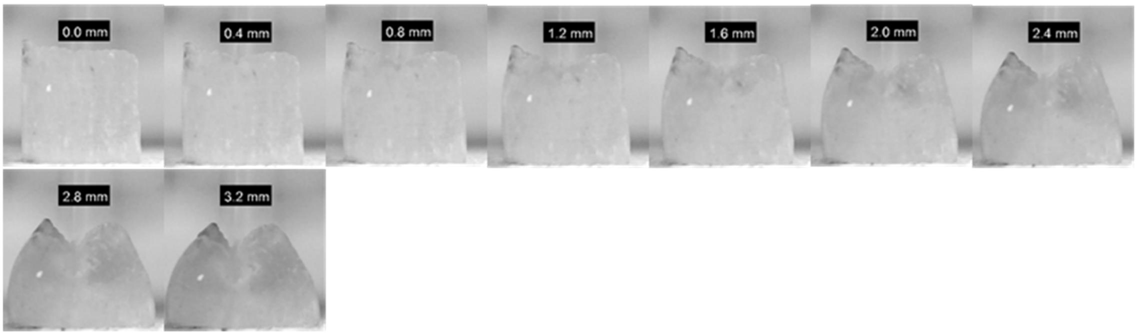

(d)

Figure S8. Micrographs taken during the compression cut test of the SH-TPU sample printed at 230 °C printing temperature and 0.8 mm infill distance. (a) XZ sample 1. (b) XZ sample 2. (c) YZ sample 1. (d) YZ sample 2.

SH-TPU_235_XZ_1

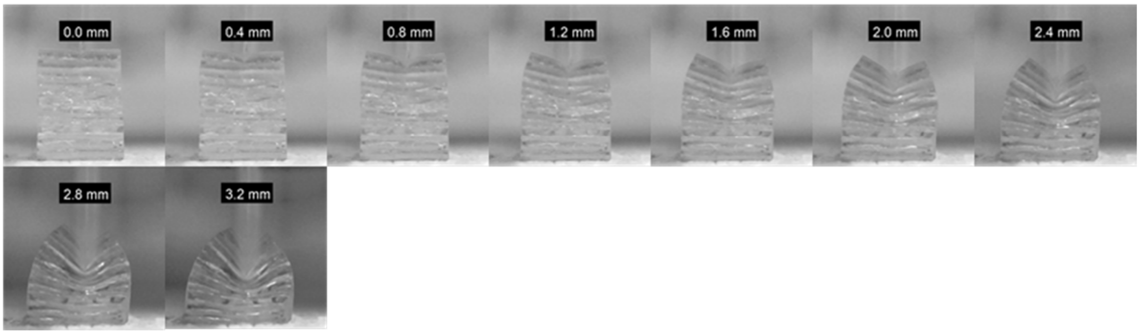

(a)

SH-TPU_235_XZ_2

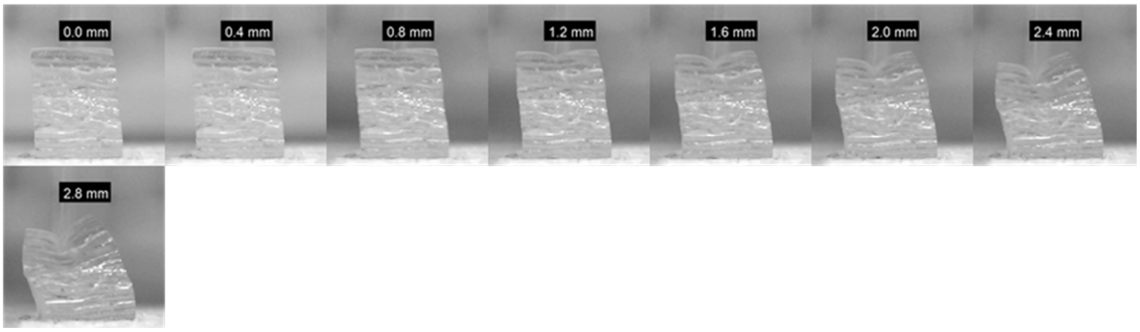

(b)

SH-TPU_235_XY_1

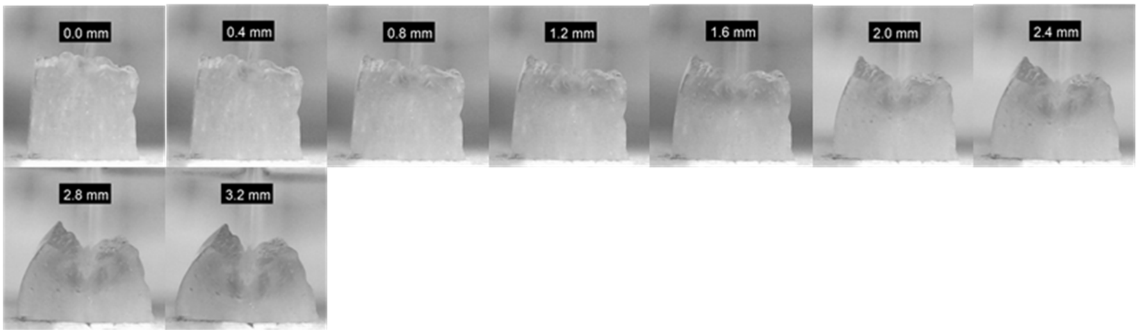

(c)

SH-TPU_235_XY_2

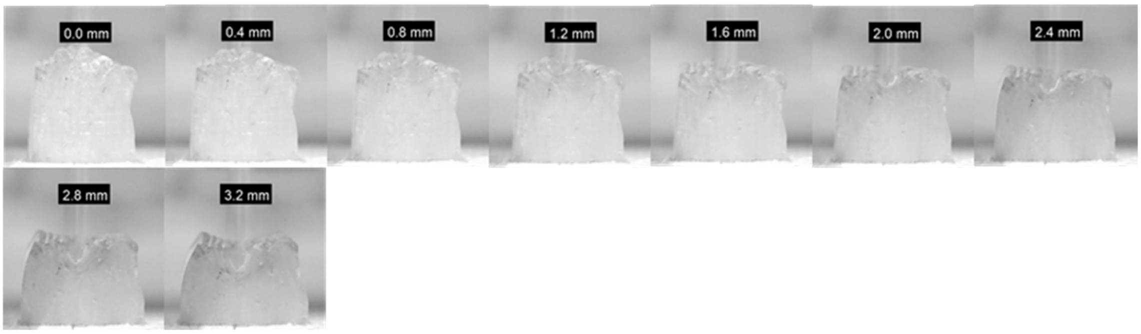

(d)

Figure S9. Micrographs taken during the compression cut test of the SH-TPU sample printed at 235 °C printing temperature and 0.8 mm infill distance. (a) XZ sample 1. (b) XZ sample 2. (c) YZ sample 1. (d) YZ sample 2.

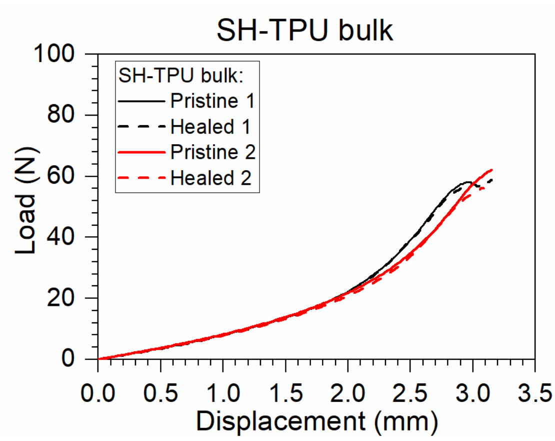

(a)

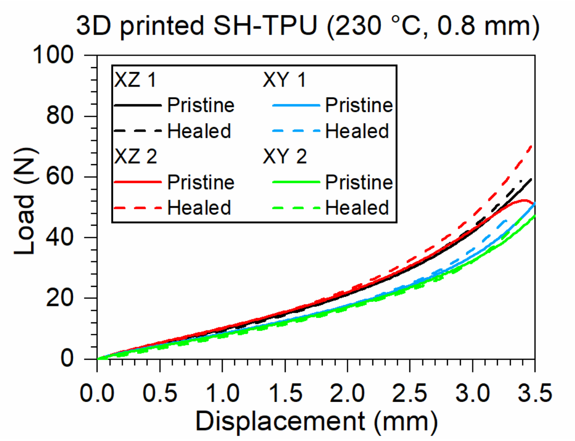

(b)

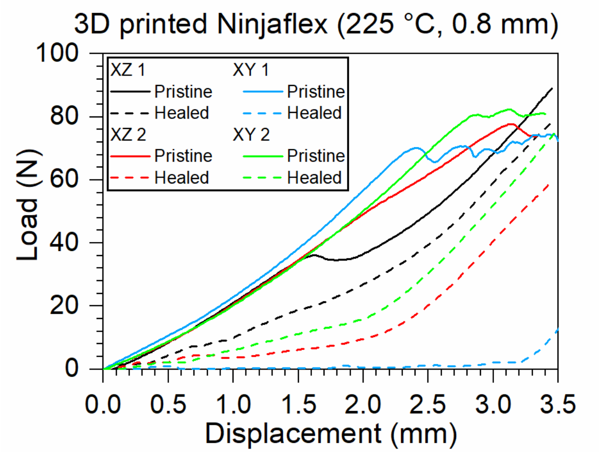

(c)

Figure S10. Force displacement curves of the compression cut test of the healing experiments. (a) Bulk SH-TPU. (b) 3D printed SH-TPU at 230 °C printing temperature and 0.8 mm infill distance. (c) 3D printed Ninjaflex at 225 °C printing temperature and 0.8 mm infill distance.

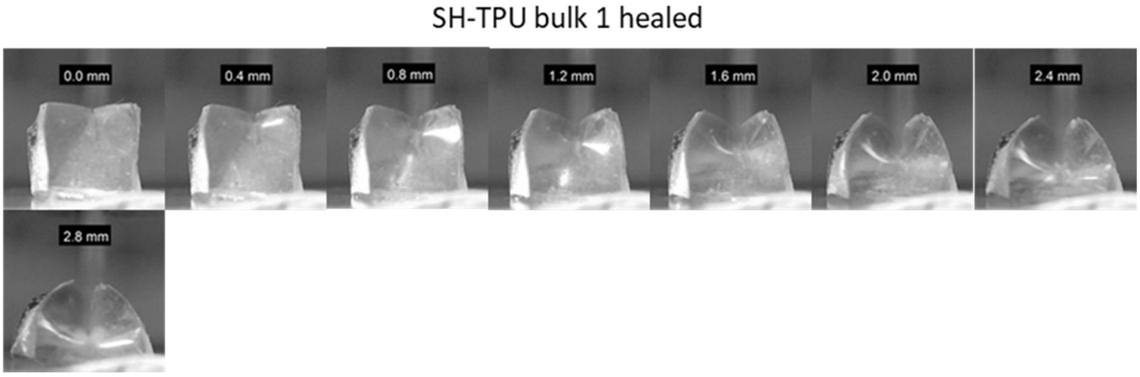

(a)

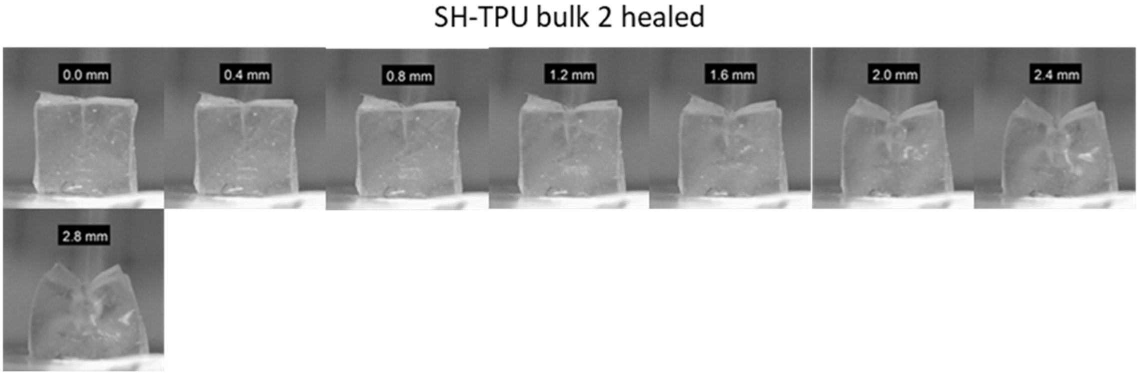

(b)

Figure S11. Micrographs taken during the compression cut test of healed bulk SH-TPU. (a) Sample 1. (b) Sample 2. For the micrographs of the pristine samples see Figure S6.

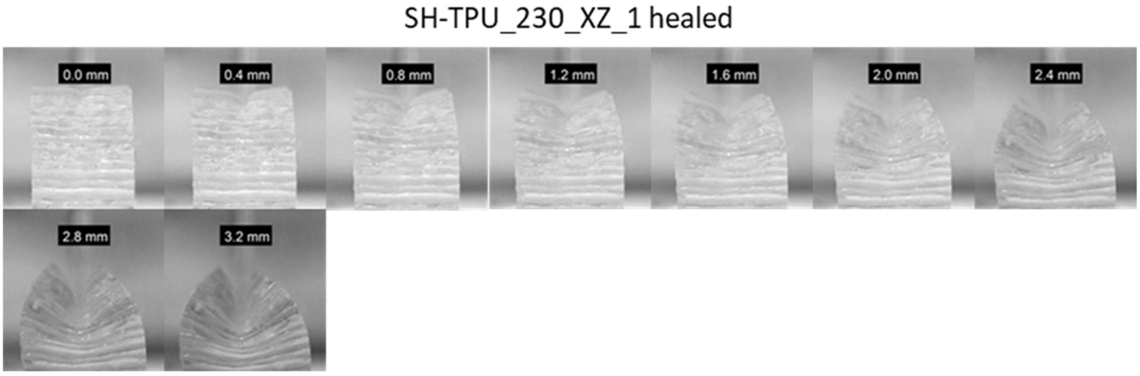

(a)

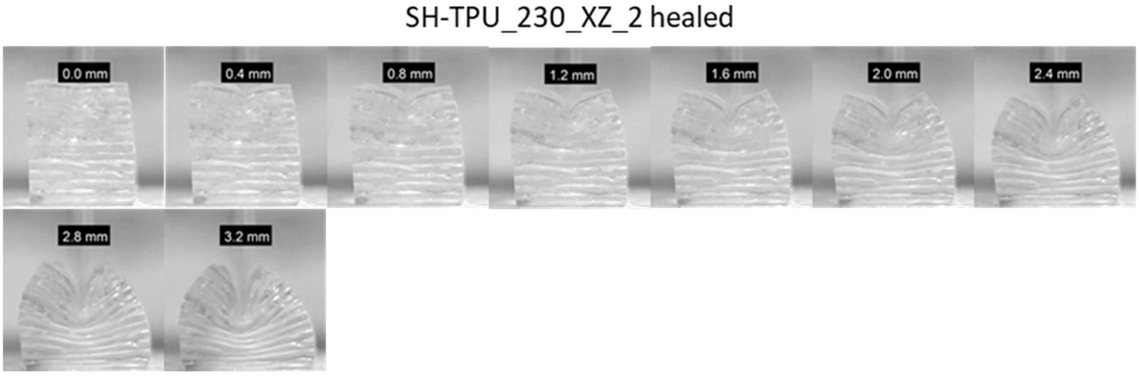

(b)

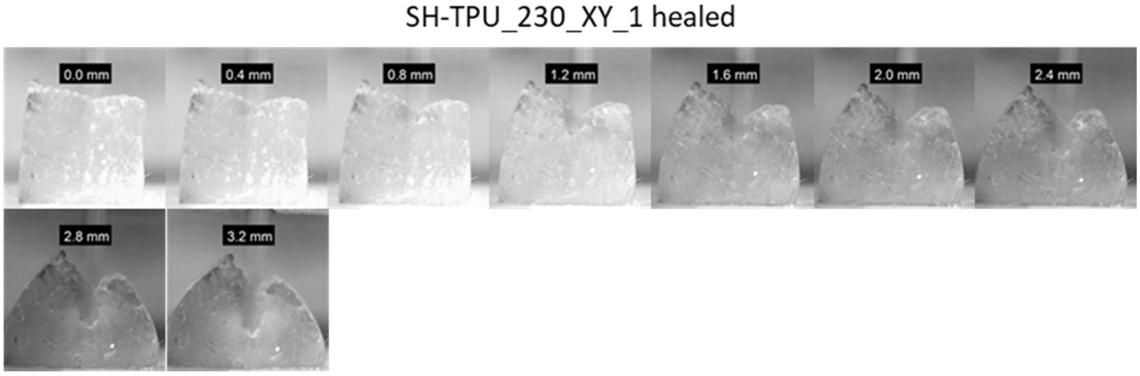

(c)

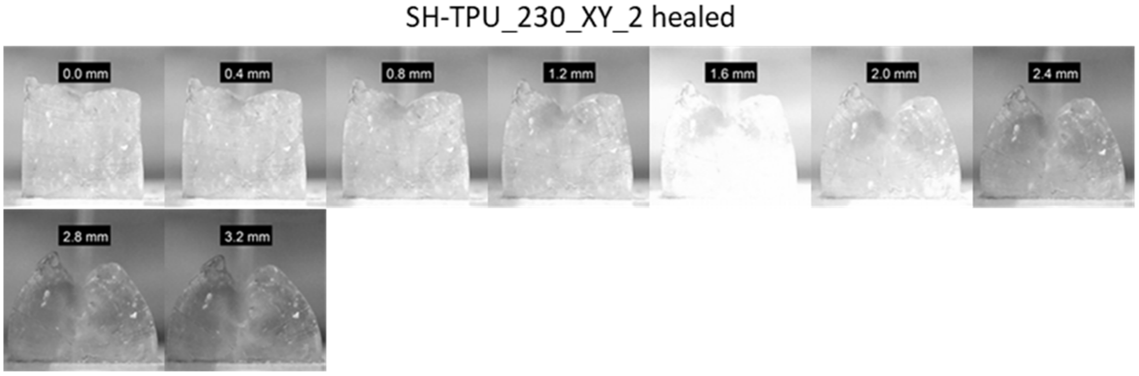

(d)

Figure S12. Micrographs taken during the compression cut test of the healed SH-TPU sample printed at 230 °C printing temperature and 0.8 mm infill distance. (a) XZ sample 1. (b) XZ sample 2. (c) YZ sample 1. (d) YZ sample 2. For the micrographs of the pristine samples see Figure S8.

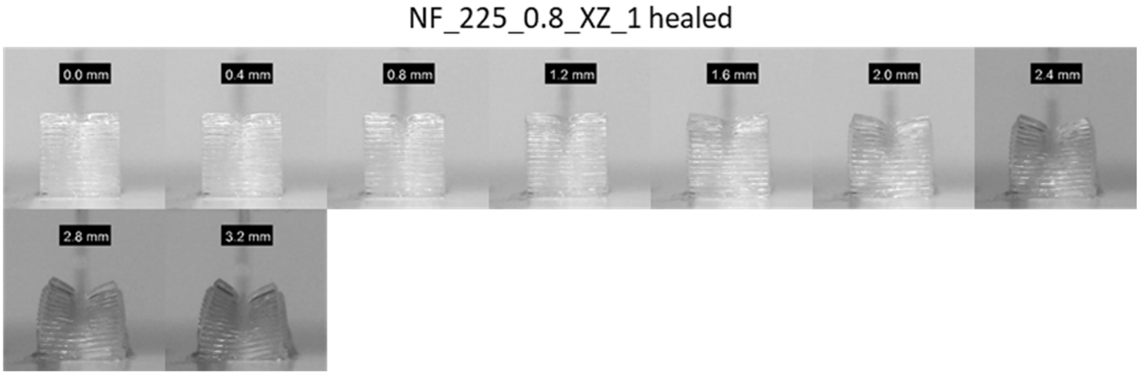

(a)

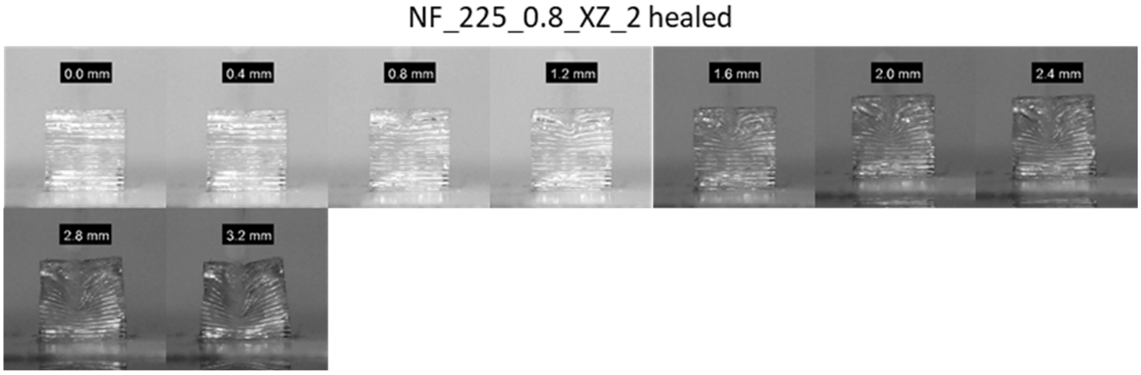

(b)

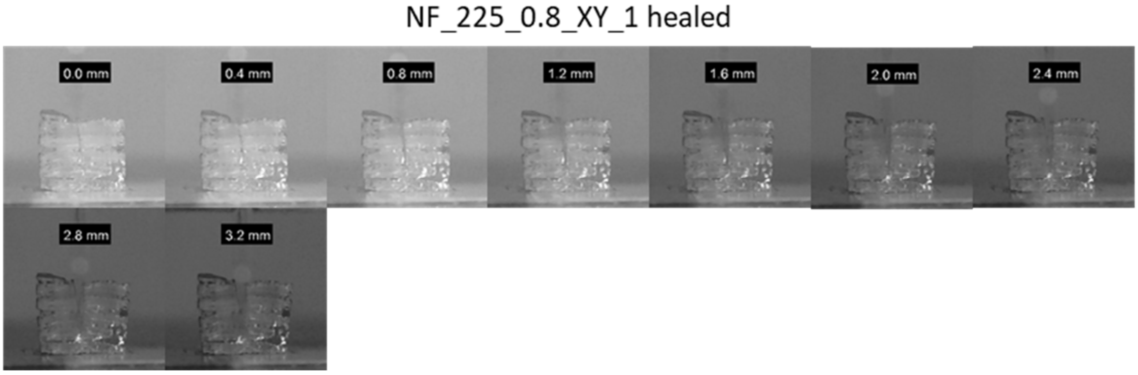

(c)

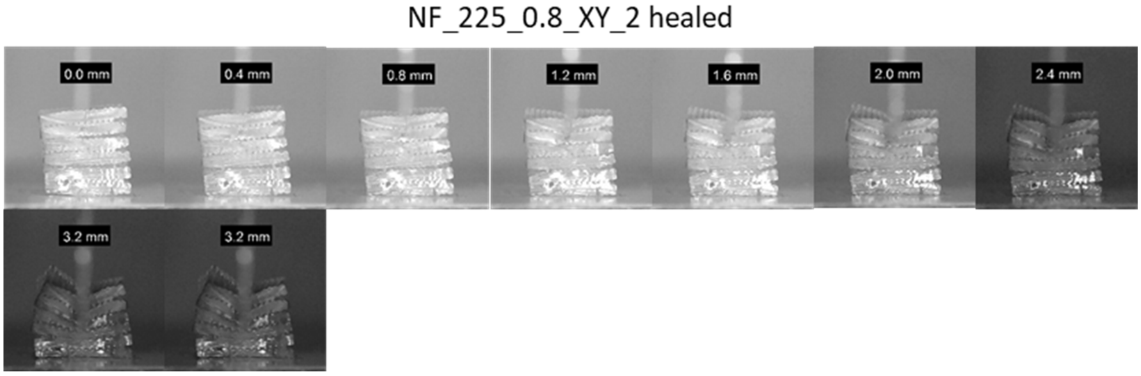

(d)

Figure S13. Micrographs taken during the compression cut test of the healed Ninjaflex sample printed at 225 °C printing temperature and 0.8 mm infill distance. (a) XZ sample 1. (b) XZ sample 2. (c) YZ sample 1. (d) YZ sample 2. For the micrographs of the pristine samples see Figure S3.

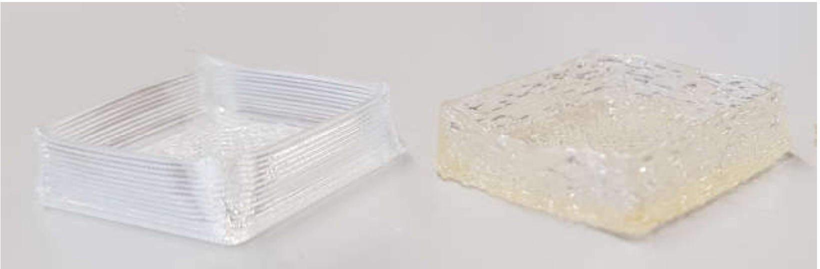

Figure S14: 3D printed hollow-vase structure with a wall of a single line. With a Ninjaflex sample on the left and a SH-TPU sample on the right (printed at 230 °C). For the vase printed with the SH-TPU, no lines of the filament used during printing are visible. However, the heterogeneity of the thickness of lab-scale SH-TPU filament resulted in an inconsistent distribution of material and thus to a poor quality wall compared to Ninjaflex. This example of 3D printing of a relatively complex structure demonstrates the possibility to not only print rectangular bars, but also more complex structures with SH-TPU.
